# CBT for Anxiety Disorders: A Practitioner Book

**DOI:** 10.1192/pb.bp.113.044867

**Published:** 2014-12

**Authors:** Roji Philip Thomas

This book includes contributions from renowned experts in the field of cognitive–behavioural therapy (CBT) for anxiety disorders such as panic disorder, agoraphobia, generalised anxiety disorder, social anxiety disorder, obsessive–compulsive disorder, post-traumatic stress disorder, specific phobias and health anxiety. It is well laid out into easy-to-read chapters, taking each of the anxiety disorders in turn and providing a clear account of the latest cognitive–behavioural models and CBT treatment methods for those conditions. In addition, there are subsections with descriptions of the diagnostic issues, epidemiology, comorbidities and pharmacological treatments. Each chapter concludes with a paragraph summarising the key points discussed.

The CBT treatment methods are covered in a great deal of detail and readers will appreciate the helpful case histories and therapist–patient dialogues in addition to the tables and figures interspersed throughout. The evidence base behind the treatment modalities is also included.

The last two chapters were particularly interesting. Chapter 8 focuses on adapting CBT techniques and making them culturally appropriate. It describes how anxiety disorders develop in people from different cultures and the authors give an illuminating insight into ‘matching the cultural characteristics of the treatment with those of the patient’ (p. 191), focusing particularly on their own experiences with using culturally adapted CBT for post-traumatic stress disorder. The final chapter gives an overview of newest entrants to the field of CBT such as acceptance and commitment therapy and mindfulness-based therapies, emphasising that newer treatments are ‘not meant to replace traditional methods of CBT but merely [refocus] attention on certain psychological processes and treatment goals’ (p. 227). The stress on improving functioning and not just symptom control is one underlying theme in the newer therapies.

Theoretical approaches in CBT have changed over the years and this book provides a comprehensive update on current theories and treatment models in this field. It will prove useful for trainees and seasoned therapists alike.

